# Proceedings of the International Workshop ‘Development of a National Diabetes Surveillance System in Germany – Core Indicators and Conceptual Framework’: Berlin, Germany. 11–12 July 2016

**DOI:** 10.1186/s12919-016-0070-5

**Published:** 2017-02-24

**Authors:** Andrea Teti, Lars Gabrys, Thomas Ziese, Christa Scheidt-Nave, Christin Heidemann, Lars Gabrys, Christian Schmidt, Andrea Teti, Ingrid-Katharina Wolf, Yong Du, Rebecca Paprott, Jens Baumert, Thomas Ziese, Christa Scheidt-Nave, Edward W. Gregg, Linda Geiss, Jan Mainz, Mette Jørgensen, Jeffrey Johnson, Fabrizio Carinci, Sarah Wild, on behalf of the Scottish Diabetes Research Network Epidemiology Group

**Affiliations:** 1 Department of Epidemiology and Health Monitoring, Robert Koch Institute, Berlin, Germany; 2 Department of Epidemiology and Health Monitoring, Robert Koch Institute, Berlin, Germany; 3 Division of Diabetes Translation, Centers for Disease Control and Prevention, Atlanta, USA; 4 Aalborg University Hospital, Psychiatry, Aalborg, Denmark; 5 University of Alberta, School of Public Health, Edmonton, Canada; 6 University of Surrey, School of Health Sciences, Guildford, UK; 7 Usher Institute of Population Health Sciences and Informatics, University of Edinburgh, Edinburgh, Scotland

## INTRODUCTION

### I1 Towards the development of a National Diabetes Surveillance system in Germany

#### Andrea Teti^†^, Lars Gabrys^†^, Thomas Ziese, Christa Scheidt-Nave

##### Department of Epidemiology and Health Monitoring, Robert Koch Institute, Berlin, Germany

###### **Correspondence:** Andrea Teti (TetiA@rki.de)

^†^equal contribution

Diabetes mellitus is a chronic metabolic disease approximately affecting 6 million adults in Germany and more than 300 million people worldwide [1]. On average, nearly 8% of adults in high-income countries and 10% in middle or low-income countries have diabetes [2]. Patients with diabetes have an increased risk for cardiovascular diseases, renal impairments, blindness, peripheral neuropathy and amputations of the lower leg and for excess mortality [3]. Unknown or insufficiently treated diabetes bears a particularly high risk for long-term complications. Diabetes is associated with a high burden of disease and costs for individuals as well as for the health care system and society as a whole. Treatment and care of diabetes led to costs of almost 48 billion euros in Germany in 2009 [4].

Against this background, the Robert Koch Institute RKI (the German National Public Health Institute) was commissioned by the Federal Ministry of Health to develop a National Diabetes Surveillance System for Germany. In order to integrate existing international expertise in the development process, the RKI organized an international workshop with experts from the U.S., Canada and Europe. The Workshop ‘Development of a National Diabetes Surveillance System in Germany – Core Indicators and Conceptual Framework’ was held in Berlin, Germany on July 11–12, 2016 and pursued the following goals: (a) to share experience on existing surveillance systems and diabetes registries (b) to refine the conceptual framework of the diabetes surveillance in Germany and for harmonization of core indictors and (c) to strengthen international collaboration. Experts were invited to present their specific experiences in planning and implementing diabetes registries or surveillance approaches (S1-S6). The conceptual framework and aims of the diabetes surveillance project in Germany were presented in the first workshop contribution by Christa Scheidt-Nave, Robert Koch-institute RKI, Berlin Germany (S1). The U.S. experience in the gradually implementation over the last 20 years of a National Diabetes Surveillance System (USDSS) was presented by Edward Gregg, epidemiologist at the CDC in Atlanta (S2). How Denmark’s health care system profits from the opportunity for quality measurement and benchmarking due to comprehensive information recorded in National Clinical Registries was introduced by Jan Mainz, member of the Danish National Board of Health (S3). The development of a National Diabetes Surveillance System (NDSS) in Canada using administrative health care data available across the country was presented by Jeffrey Johnson from the School of Public Health at the University of Alberta (S4). The international approach of using Health Care Quality Indicators (HCQI) for diabetes at the European level and within the OECD Health Care Quality Indicator Project was presented by Fabrizio Carinci, from the School of Health Sciences in Surrey, UK (S5). Sarah Wild from the Scottish Diabetes Research Network introduced the population-based Scottish national diabetes register, which allows daily electronic capture of data from primary and secondary health care (S6).

**References**

1. Heidemann C, Du Y, Schubert I, Rathmann W, Scheidt-Nave C (2013). Prevalence and temporal trend of known diabetes mellitus. Results of the German Health Interview and Examination Survey for Adults (DEGS1). Bundesgesundheitsblatt 56:668–677. doi:10.1007/s00103-012-1662-5.

2. Scully T (2012). Diabetes in numbers. Nature, 485(7398), S2-S3. doi:10.1038/485S2a.

3. ADA (2012). Executive summary: Standards of medical care in diabetes-2012. American Diabetes Association ADA. Diabetes Care, 35(1):4–10. doi:10.2337/dc12-s004.

4. Koster I, Schubert I, Huppertz E (2012). [Follow up of the CoDiM-Study: Cost of diabetes mellitus 2000–2009]. Deutsche Medizinische Wochenschrift, 137(19): 1013–1016. doi:10.1055/s-0032-1304891.

## SPEAKERS PRESENTATIONS

### S1 Diabetes Surveillance in Germany – Status quo and Perspectives

#### Christin Heidemann, Lars Gabrys, Christian Schmidt, Andrea Teti, Ingrid-Katharina Wolf, Yong Du, Rebecca Paprott, Jens Baumert, Thomas Ziese, Christa Scheidt-Nave

##### Department of Epidemiology and Health Monitoring, Robert Koch Institute, Berlin, Germany

###### **Correspondence:** Christa Scheidt-Nave (Scheidt-NaveC@rki.de)

Over the last years, several programs and action plans targeted at the prevention and control of diabetes were initiated on the international [1–2] and national [3–7] level. In 2015, the German Ministry of Health funded a four year research project to evaluate the feasibility of a National Diabetes Surveillance System in Germany under the lead of the Robert Koch Institute. The aims of the Surveillance System are (1) to build a comprehensive and sustainable database for Public Health and Health Services Research on diabetes in line with internationally agreed indicators, (2) to identify and overcome barriers (e.g. technical, economical, legal, ethical, political barriers) to data sharing across health sectors, establish a surveillance at the national and the regional level, (3) to enable evidence-informed policy advising, and (4) to make diabetes a paradigm for non-communicable disease surveillance.

In lack of a legal basis for the use of unified patient identifiers, the framework is conceptualized as a Public Health Surveillance System that is based on available data from nationwide health surveys of the Robert Koch Institute as well as secondary data sources from different health sectors and research settings (e.g. type 1 diabetes registries for children and adolescents; information system for health care data ‘data transparency’; Disease Management Programs on diabetes; Diagnosis Related Groups statistics for hospital admissions). This large but fragmented database will be assessed with regard to sustainable monitoring based on selected diabetes core indicators. A preliminary set of core indicators was identified based on a review of indicators used in established diabetes surveillance systems and diabetes registries in other countries as well as indicators relevant to the German Disease Management Programs for type 1 and type 2 diabetes. These core indicators can be assigned to the following four areas of action: 1) ‘Reducing diabetes risk’, 2) ‘Improving diabetes diagnosis and treatment’, 3) Reducing diabetes complications’, and 4) ‘Reducing the burden of disease and costs associated with diabetes’. Sociodemographic variables considered essential for stratified analyses were added. The initial indicator list was reviewed by national and international experts with respect to their relevance (essential, important, additional, or negligible). A continued collaboration is intended for refining the conceptual framework and harmonization of core indictors.

**References**

1. The Saint Vincent Declaration on diabetes care and research in Europe. Acta Diabetologia. 1989(10): 143–144.

2. Zimmet PZ, Magliano DJ, Herman WH, Shaw JE (2014). Diabetes: a 21st century challenge. Lancet Diabetes Endocrinol 2(1):56–64. doi:10.1016/S2213-8587(13)70112-8.

3. Weikert B, Weinbrenner S, Meyerrose B, Abholz HH, Haller N, Kopp I, et al. (2011). [German National Disease Management Guidelines NDMG. Evidence-based support for decision making in type 2 diabetes in the German Healthcare System]. Diabetes aktuell 2011(9):70–74.

4. Fuchs S, Henschke C, Blümel M, Busse R (2014). Disease management programs for type 2 diabetes in Germany. Dtsch Ärztebl Int( 111):453–463. doi:10.3238/arztebl.2014.0453

5. Federal Ministry of Health (2016). Defining national health goals. Available from: http://www.bmg.bund.de/en/health/national-health-goals.html, accessed November 4, 2016.

6. Kleinwechter H, Schäfer-Graf U, Bührer C, Hoesli I, Kainer F, Kautzky-Willer A, Pawloski B, Schunck K, Somville T, Sorger M (2014). Gestational diabetes mellitus (GDM) diagnosis, therapy and follow-up care. Exp Clin Endocrinol Diabetes, 122(07):395–405. doi:10.1055/s-0034-1366412.

7. Federal Ministry of Health (2016). The Preventive Health Care Act (2015). Available from http://www.bmg.bund.de/en/prevention/the-preventive-health-care-act.html, accessed November 4, 2016.

## SPEAKERS PRESENTATIONS

### S2 The U.S. Diabetes Surveillance System: Lessons and Applications for Health Policy

#### Edward W. Gregg, Linda Geiss

##### Division of Diabetes Translation, Centers for Disease Control and Prevention, Atlanta, USA

###### **Correspondence:** Edward Gregg (edg7@cdc.gov)

Chronic disease surveillance systems play several critical roles in the population-wide reduction of chronic diseases. Surveillance systems ideally provide efficient monitoring of risk factors, care, the delivery of preventive interventions, prevalence and incidence, and morbidity, all of which are applied across sub-segments of the population, and according to geographic location. When such data is further integrated with findings from intervention effectiveness research and health impact and economic modeling, it forms the basis for effective public health decision making and to optimally prioritize approaches and populations for prevention. The U.S. National Diabetes Surveillance System (USDSS) was gradually built as an integrated, multi-component composition of surveys and registries [1]. Primary information on risk factors, prevalence, and incidence comes from the National Health Interview Survey (NHIS), Behaviour Risk Factor Surveillance System (BRFSS), and National Health and Nutrition Examination Surveys (NHANES). Data on diabetes-related morbidity is primarily derived from the National Hospital Discharge Survey (NHDS), National Inpatient Sample (NIS), and specialized registries like the U.S. Renal Data System, while mortality estimates are estimated using vital statistics data that are linked to population survey data. Specialized registries such as the SEARCH Study are also used to assess specific problems such as diabetes in youth [2]. The system relies largely on a design of serial cross-sections, permitting both yearly estimates as well as multi-year aggregates. By incorporating the USDSS into multi-level and Markov modelling, the data are also used to examine specific geographic variation, and for the estimation of lifetime risk, disability-free life years lost due to specific conditions, and for cost-effectiveness modelling of candidate interventions. Finally, the system has developed practical, interactive data visualization tools to help leaders and policy makers in public health. Over the past 20 years, the USDSS has documented several phases and diverse sub-trends in the population, including the growth of prevalence and incidence and lifetime risk, improvements in care, and reductions in diabetes-related complications and mortality, and has highlighted important variation in all indicators and diversification of morbidity that have provided the basis for focused public health resources and interventions for diabetes [3, 4, 5]. The key surveillance challenges for the future include improving the geographic texture of measurement of risk, care, and control, improving assessment of primary prevention behaviors and interventions, improving assessment of risks and adverse events, incorporating electronic medical records and longitudinal measurements into surveillance of care, and application of NDSS data into assessment of natural experiments of health policy interventions. Finally, better standardization of systems will facilitate multi-national comparisons and better international pooling studies to better tackle the global problem of diabetes.

**References**

1. Centers for Disease Control and Prevention (2016). National Diabetes Surveillance System. Available from http://www.cdc.gov/diabetes/statistics/index.htm, accessed November 4, 2016.

2. Dabelea D, Mayer-Davis EJ, Saydah S, Imperatore G, Linder B et al. (2014). Prevalence of type 1 and type 2 diabetes among children and adolescents from 2001 to 2009. JAMA, 311(17):1778–1786. doi:10.1001/jama.2014.3201

3. Geiss LS, Wang J, Cheng YJ, Thompson TJ, Barker L, Li Y, Albright AL, Gregg E (2014). Prevalence and Incidence trends for diagnosed diabetes among adults aged 20 to 79 years, United States, 1980–2012. JAMA 24; 312(12):1218–1226. doi:10.1001/jama.2014.11494.

4. Ali MK, Bullard KM, Saaddine JB, Cowie CC, Imperatore G, Gregg EW (2013). Achievement of goals in US diabetes care, 1999–2010. New England Journal of Medicine, 368(17):1613–1624. doi:10.1056/NEJMsa1213829.

5. Gregg EW, Li Y, Wang J, Rios Burrows N, Ali MK, Rolka D et al. (2014). Changes in diabetes-related complications in the United States, 1990–2010. New England Journal of Medicine, 370(16):1514–1523. doi:10.1056/NEJMoa1310799.

### S3 Quality Improvement using Performance and Outcome Measurement: Experiences from Denmark

#### Jan Mainz, Mette Jørgensen

##### Aalborg University Hospital, Psychiatry, Aalborg, Denmark

###### **Correspondence:** Jan Mainz (jan.mainz@rn.dk)

Denmark has unique opportunities for quality measurement and benchmarking due to comprehensive information recorded in the National Clinical Registries. This presentation clarifies the use of these registries to improve the quality of care provided by the Danish health care system.

The public and mainly tax-financed Danish health care system provides universal health coverage to the country’s 5.5 million inhabitants. It is a stated priority, that all citizens should have free and equal access to health care services [1]. By the use of a unique, ten-digit civil registration number assigned to all Danish residents, each contact with the health care system is recorded in National Clinical Registries. Currently 61 disease-specific registries systematically document and develop the quality of care provided by the Danish health care system by the use of process-, and outcome indicators in accordance with national clinical guidelines [2, 3]. Reporting is mandatory for all public hospitals in Denmark ensuring a high coverage > 90%. Clinicians and managers at the hospital department and clinics receive continuous feedback of their adherence to the recommended clinical guidelines. Structured audits are further initiated on national, regional and local basis to ensure implementation of improvements whilst all data are published yearly to inform the public of the development in the quality of care.

Nationwide studies demonstrate significant improvements since the initiation of systematic indicator monitoring combined with continuously auditing in the Danish health care system. In this regard, the adherence to several guidelines recommended process indicators increased between 2004 and 2011 for patients hospitalized with schizophrenia and between 2003 and 2010 for incident heart failure patients seen at hospital departments and outpatient clinics [4, 5]. Similar efficiency have been shown in the quality of care for stroke, high volume cancers, coronary percutaneous coronary intervention/coronary artery bypass surgery, hernia, childhood diabetes and perforated gastric ulcer in Denmark. Nevertheless, variation in the delivered quality of care remains between both the specific disease and the hospital departments. In conclusion, the quality of care has improved substantially since systematic monitoring and auditing was initiated in Denmark.

**References**

1. OECD (2013). OECD Reviews of Health Care Quality: Denmark: Raising Standards. Organisation for Economic Co-operative and Development, Paris. doi:10.1787/9789264191136-en

2. Mainz J, Krog BR, Bjornshave B, Bartels P (2004). Nationwide continuous quality improvement using clinical indicators: the Danish National Indicator Project. Int J Qual Health Care, 16:i45-i50

3. Mainz J, Kristensen S, Bartels P (2015). Quality improvement and accountability in the Danish health care system. International Journal for Quality in Health Care, 27(6): 523–527. doi:10.1093/intqhc/mzv080

4. Jørgensen M, Mainz J, Svendsen ML, Nordentoft M, Voldsgaard I, Baandrup L, Bartels P, Johnsen SP (2015). Improving quality of care among patients hospitalized with schizophrenia: A nationwide initiative. BJPsych Open, 1(1): 48–53. doi:10.1192/bjpo.bp.115.000406

5. Nakano A, Johnsen SP, Frederiksen BL, Svendsen ML, Agger C, Schjødt I, Egstrup K (2013). Trends in quality of care among patients with incident heart failure in Denmark 2003–2010: a nationwide cohort study. BMC Health Services Research, 13(1):391. doi:10.1186/1472-6963-13-391

### S4 From Diabetes Surveillance to Chronic Disease Surveillance: Experience from a Canadian Perspective

#### Jeffrey Johnson (jeff.johnson@ualberta.ca)

##### University of Alberta, School of Public Health, Edmonton, Canada

Using administrative health care utilization data available in all provinces and territories across the country, Canada developed a National Diabetes Surveillance System (NDSS) build on federal/provincial/territorial (F/P/T) partnerships. A series of pilot studies initially established the validity of a case definition for diabetes based on ICD9 billing codes. The Canadian NDSS reported trends in diabetes incidence and prevalence in a federated model, coordinated federally by the Public Health Agency of Canada, with each of the P/T contributing aggregated surveillance data, generated using a common computing syntax. Building on this model the PHAC sponsored work to develop and test case definitions for other chronic conditions, and the NDSS evolved into the Canadian Chronic Diseases Surveillance System (CCDSS), with ongoing surveillance for diabetes, hypertension, selected mental illnesses, and chronic respiratory diseases. National pilot studies have been conducted for heart disease and musculoskeletal conditions. Methodological challenges for the CCDSS include lack of completeness of disease case capture in physician billing claims, poor measurement validity of diagnosis codes for some chronic conditions, and striking a balance between reporting all cases with interpretable rates and following the latest residual disclosure guidelines. Further, the NDSS/CCDSS are limited to surveillance of disease incidence, prevalence and mortality. Further developments are needed, and ongoing in some P/T jurisdictions, to build on the established administrative data sources to embellish the available information, and extend surveillance to individual level risk factors, health status and behaviours. Such efforts would link survey data to administrative data sources to provide more comprehensive risk factor and disease surveillance.

**References**

1. Public Health Agency of Canada (2009). Report from the National Diabetes Surveillance System: Diabetes in Canada 2009. Public Health Agency of Canada, Ottawa. Available at: http://www.phac-aspc.gc.ca/publicat/2009/ndssdic-snsddac-09/index-eng.php, accessed November 4, 2016.

2. Public Health Agency of Canada (2011). Diabetes in Canada: Facts and figures from a public health perspective. Public Health Agency of Canada, Ottawa 2011. Available at: http://www.phac-aspc.gc.ca/cd-mc/publications/diabetes-diabete/facts-figures-faits-chiffres-2011/index-eng.php, accessed November 4, 2016.

3. Government of Canada (2016), Canadian Chronic Disease Surveillance System, 1996/97-2011/2012. Government of Canada. Available at: http://open.canada.ca/data/en/dataset/9525c8c0-554a-461b-a763-f1657acb9c9d, accessed November 4, 2016.

4. Betancourt MT, Roberts KC, Bennett T-L, Driscoll ER, Jayaraman G, Pelletier L (2014). Monitoring chronic diseases in Canada: the Chronic Disease Indicator Framework. Chronic Dis Inj Can.; 34(1)1:1–30.

5. Al Sayah F, Majumdar SR, Soprovich A, Wozniak, Johnson L, Qiu ST, Rees W, Johnson JA (2015). The Alberta's Caring for Diabetes (ABCD) Study: Rationale, Design and Baseline Characteristics of a Prospective Cohort of Adults with Type 2 Diabetes. Can J Diabetes. 39(3):113–119. doi: 10.1016/j.jcjd.2015.05.005.

### S5 Diabetes Health Care Quality Indicators (HCQI) – An international Approach

#### Fabrizio Carinci (f.carinci@surrey.ac.uk)

##### University of Surrey, School of Health Sciences, Guildford, UK

Continuous monitoring of health care quality indicators (HCQIs) is increasingly seen as an effective solution to steer governments towards high performing health systems. Comparative analysis may help identifying areas for immediate improvement through mutual collaboration within organizations e.g. the Organization for Economic Co-operation and Development (OECD) and the European Union (EU).

The OECD Framework of Health Systems Performance is a multidimensional matrix adopted for regular publication by 34 Member States (MS) [1] in which HCQIs have been allocated in a multidimensional quality matrix measuring efficiency, safety and responsiveness along the life cycle of the individual.

Targeted OECD studies have been conducted to improve HCQIs that were considered too variable across countries e.g. amputation rates in diabetes [2]. New definitions were released for Health at a Glance 2015 [3] , where Germany showed a rate of 9.8 major amputations x100,000 total population, third highest after Israel and Slovenia out of 17 countries (median: 6.0; range: 1.07-17.05).

Amputations in diabetes pose fundamental questions for public health monitoring: are this data accurate? Is there a problem of heterogeneity of data definitions, or instead, are we facing a real clinical problem on which governments should act? Answers may not be straightforward without a system in place and indeed, the data delivered to the OECD are officially provided by the government.

During the last decade, the European Union sponsored specific initiatives e.g. the EUBIROD project (www.eubirod.eu) to create a common EU infrastructure for diabetes monitoring. The project delivered standardized definitions, privacy rules and the software required to make it happen [4].

Developing a national surveillance system for a large country e.g. Germany may be not less challenging than carrying out diabetes reporting across Europe. A common system would allow: a) saving financial resources by avoiding duplications while exploiting the existing information infrastructure; b) overcoming data protection issues through the development of a platform that would implement “privacy by design”; c) automatic updating and immediate testing of new definitions.

The EUBIROD project provided useful criteria that may show how to proceed in a sustainable direction, adopting a “bazaar model” rather than proposing a new “cathedral”. Such an approach would allow reconciling technical aspects with the need to resolve clinical questions of utmost importance for all citizens. To proceed further, Germany may need to define the essential level of information first and then implement the national infrastructure accordingly [5]. Other countries may directly benefit from the result of such a strategic initiative.

**References**

1. Carinci F, Van Gool K, Mainz J, Veillard JH, Pichora E, Januel JM, Arispe I, Kim SM, and Klazinga NS on behalf of the OECD Health Care Quality Indicators Expert Group, Towards actionable international comparisons of health system performance: expert revision of the OECD framework and quality indicators, International Journal for Quality in Health Care, Apr; 27(2):137–46. doi: 10.1093/intqhc/mzv004.

2. Carinci F, Massi Benedetti M, Klazinga NS, Uccioli, L. (2016). Lower extremity amputation rates in people with diabetes as an indicator of health systems performance. A critical appraisal of the data collection 2000–2011 by the Organization for Economic Cooperation and Development OECD. Acta Diabetologica, 53(5), 825–832. doi:10.1007/s00592-016-0879-4.

3. OECD (2015). Health at a Glance 2015. OECD Indicators. OECD Publishing, Paris. doi:10.1787/health_glance-2015-en.

4. Cunningham SG, Carinci F, Brillante M, Leese GP, McAlpine RR, Azzopardi J, Beck P, Bratina N, Boucquet V, Doggen K, Jarosz-Chobot PK, Jecht M, Lindblad U, Moulton T, Metelko Ž, Nagy A, Olympios G, Pruna S, Skeie S, Storms F, Di Iorio CT, Massi Benedetti M (2016). Defining a European Diabetes Data Dictionary for Clinical Audit and Healthcare Delivery. Core Standards of the EUBIROD project. Methods of Information in Medicine, 55(2): 166–176. doi:10.3414/ME15-01-0016.

5. Carinci F (2015). Essential levels of health information in Europe: An action plan for a coherent and sustainable infrastructure. Health Policy, 119(4):530–538. doi:10.1016/j.healthpol.2014.11.016.

### S6 Diabetes indicators and outcomes. Insights from the Scottish diabetes register and data linkage

#### Sarah Wild, on behalf of the Scottish Diabetes Research Network Epidemiology Group

##### Usher Institute of Population Health Sciences and Informatics, University of Edinburgh, Edinburgh, Scotland

###### **Correspondence:** Sarah Wild (Sarah.Wild@ed.ac.uk)

The need to develop plans to prevent, identify and treat diabetes and its complications at local, national and regional levels and the setting of targets for Europe was first formally identified in the 1989 St Vincent declaration on the treatment of diabetes. Progress has been made but full implementation has not been achieved and diabetes prevalence continues to rise, along with increasing individual and societal costs of complications. A national population-based register of people with a diagnosis of diabetes was set up in Scotland following agreement of a core dataset and development of earlier local registers. The register is populated by daily electronic capture of data from primary and secondary care and includes demographic, clinical, laboratory, eye screening and prescribing information and a patient portal. The register became comprehensive following the introduction of a pay-for-performance scheme for general practitioners in the United Kingdom, the Quality and Outcomes Framework [QOF] in 2004. Prevalence of diagnosed diabetes in 2015 was 5.3% in the population of Scotland of all ages of which approximately 90% was type 2 diabetes). Higher prevalence is observed in men than women, with increasing age (to a maximum of 17% among men of 75–79 years of age) and a marked inverse socio-economic gradient (prevalence among women in the most-deprived fifth of the population is double that of women in the least deprived fifth of the population) [1]. Age-standardised incidence of type 2 diabetes has declined in the last decade but prevalence has increased due to increasing survival [2]. Data linkage to hospital admission, cancer registration, mortality and other disease registers is possible with permission from the national Public Benefit and Privacy Panel. Examples of analyses of linked data includes the following findings: that hospital records under-estimate diabetes prevalence by over 40% [3], that type 2 diabetes is associated with approximately 50% higher mortality than the general population[1], that type 1 diabetes is associated with the loss of about 12 years of life expectancy among 20 year olds [4] and that relative risks of cancer associated with diabetes are similar to those observed for obesity [5]. Approaches to improving outcomes for people with diabetes have been addressed in the Scottish Government’s recent Diabetes Improvement Plan

**References**

1. Walker JJ, Livingstone SJ, Colhoun HM, Lindsay RS, McKnight JA, Morris AD, Petrie JR, Philip S, Sattar N, Wild SH (2011). Effect of Socioeconomic Status on Mortality Among People With Type 2 Diabetes. A study from the Scottish Diabetes Research Network Epidemiology Group, 34(5): 1127–1132. doi:10.2337/dc10-1862.

2. Read SH, Kerssens JJ, McAllister DA, Colhoun HM, Fischbacher CM, Lindsay RS, McKnight J, Wild SH (2016). Trends in type 2 diabetes incidence and mortality in Scotland between 2004 and 2013. Diabetologia, 59(10), 2106–2113. doi:10.1007/s00125-016-4054-9.

3. Anwar H, Fischbacher CM, Leese G, Lindsay R, McKnight J, Wild SH (2011). Assessment of the under-reporting of diabetes in hospital admission data: a study from the Scottish Diabetes Research Network Epidemiology Group: Under-reporting of diabetes in hospital admission data. Diabetic Medicine : a journal of the British Diabetic Association, 28(12):1514–1519. doi:10.1111/j.1464-5491.2011.03432.x.

4. Livingstone SJ, Levin D, Looker HC, Lindsay RS, Wild SH, Joss N, Leese G, Leslie P, McCrimmon RJ, Metcalfe W et al. & et al. (2015). Estimated life expectancy in a scottish cohort with type 1 diabetes, 2008–2010. JAMA, 313(1):37–44. doi:10.1001/jama.2014.16425.

5. Walker JJ, Brewster DH, Colhoun HM, Fischbacher CM, Leese GP, Lindsay RS, McKnight JA, Philip S, Sattar N, Stockton DL, Wild SH (2013). Type 2 diabetes, socioeconomic status and risk of cancer in Scotland 2001–2007. Diabetologia, 56(8), 1712–1715. doi:10.1007/s00125-013-2937-6.

**Declarations**

**Funding**

Publication fees are covered by Robert Koch-Institute, Berlin Germany. The author(s) received no financial support for the research, authorship, and/or publication of this article.

**Competing interests**

The author(s) declared no potential conflicts of interest with respect to the research, authorship, publication, and/or publication of this article.

**Consent for publication**

Not applicable

**Ethics approval and consent to participate**

Not applicable

